# Associations between utilization rates and patients’ health: a study of spine surgery and patient-reported outcomes (EQ-5D and ODI)

**DOI:** 10.1186/s12913-020-4968-2

**Published:** 2020-02-22

**Authors:** Jan Håkon Rudolfsen, Tore K. Solberg, Tor Ingebrigtsen, Jan Abel Olsen

**Affiliations:** 1 Department of Community Medicine, UiT – The Arctic University of Norway, Tromsø, Norway; 2 Department of Neurosurgery, University Hospital of Northern Norway, and the Norwegian Registry for Spine Surgery (NORspine), Tromsø, Norway; 3 Department of Clinical Medicine, UiT - The Arctic University of Norway, Tromsø, Norway; 4 Division of Health Services, Norwegian Institute of Public Health, Oslo, Norway; 5 Centre for Health Economics, Monash University, Melbourne, Australia

**Keywords:** Regional variation, Baseline health, Health gain, EQ-5D, ODI, Flat of the curve

## Abstract

**Background:**

A vast body of literature has documented regional variations in healthcare utilization rates. The extent to which such variations are “unwarranted” critically depends on whether there are corresponding variations in patients’ needs. Using a unique medical registry, the current paper investigated any associations between utilization rates and patients’ needs, as measured by two patient-reported outcome measures (PROMs).

**Methods:**

This observational panel study merged patient-level data from the Norwegian Patient Registry (NPR), Statistics Norway, and the Norwegian Registry for Spine Surgery (NORspine) for individuals who received surgery for degenerative lumbar spine disorders in 2010–2015. NPR consists of hospital administration data. NORspine includes two PROMs: the generic health-related quality of life instrument EQ-5D and the disease-specific, health-related quality of life instrument Oswestry Disability Index (ODI). Measurements were assessed at baseline and at 3 and 12 months post-surgery and included a wide range of patient characteristics. Our case sample included 15,810 individuals. We analyzed all data using generalized estimating equations.

**Results:**

Our results show that as treatment rates increase, patients have better health at baseline. Furthermore, increased treatment rates are associated with smaller health gain.

**Conclusion:**

The correlation between treatment rates and patients health indicate the presence of unwarranted variation in treatment rates for lumbar spine disorders.

## Background

Systematic variations in the utilization rates of healthcare services are well established and apparent in all developed healthcare systems [1, 2]. Variations are not inherently bad, and variations due to fluctuations in patients’ need for treatment are considerd as *warranted variations*. However, empirical findings demonstrate how they result from factors unrelated to patients’ need for treatment – i.e. *unwarranted variations* [3]. Based on aggregate data, earlier studies demonstrated how healthcare services exhibit diminishing returns [4–6], a phenomenon commonly known as “flat of the curve” [7]. However, evidence for specific conditions is scarce.

Wennberg suggested a framework for analysis of variation in population based treatment rates that has been widely adopted [8]. The framework categorized variation as being present in either (i) “effective care,” (ii) “preference-sensitive care,” and (iii) “supply-sensitive care”. Effective care refers to interventions with few treatment options, for which benefits far outweigh risk and the optimal rate of utilization is 100% of patients who need treatment according to evidence-based guidelines. Care is deemed preference-sensitive when diagnostic test results are open to interpretation and two or more generally accepted treatment options are available. Variations will reflect systematic differences in patients’ or physicians’ preferences. Supply-sensitive care comprises activities for which the frequency of use depends on the capacity of the local healthcare system (e.g., hospital beds, diagnostic equipment, or physicians). At an aggregate level, variations in surgery for degenerative disorders of the spine might exchibit variation from all three categories.

Patients with degenerative disorders of the spine report significant reduction in health-related quality of life (HRQoL). Low back and neck pain and are the largest contributors to health loss in Norway [9]. Such disorders represent the largest single cause of sick leave worldwide (11% in Norway, estimated social cost of 1–1.6 billion euro) [10, 11]. These disorders can be treated conservatively or with surgery. In some cases surgery is clearly effective [12], but preferences and supply sensitivity may explain why treatment rates differ.

Related studies, considering the association between patients’ need and treatment rates tend to use mortality or readmission rates [13–15]. Although such measures are objective, easily obtainable, and arguably can be used as a proxy for health or quality of care, they are inadequate when considering variations in specific elective treatments where *unwarranted variations* are likely to excist [16]. Further, they do not reflect patients’ need for treatment. When patients’ need is not a matter of either/or, but rather of different degrees, a continuous assessment of health is more suitable, whereby patients report their level of discomfort using patient-reported outcome measures (PROMs).

This paper considered HRQoL at baseline and post-treatment in relation to treatment rates. Our unique dataset was retreived from both administrative and medical registries for patients who underwent surgery for lumbar disc herniation (LDH) or lumbar spinal stenosis (LSS). A sample representative of the treated population demonstrates how need (i.e., “ill health” and “capacity to benefit”) varied across hospital regions. We show how such differences are associate with regional variation in treatment rates.

Under Norway’s public health insurance scheme, patients are eligible for free specialized care and surgeons are instructed to prioritize care in accordance with official guidelines. Hence, preference or supply should reflect both regional treatment rates and patients’ health. The hypothesis presented here is simply: in regions with high (low) treatment rates, surgeons’ perceived threshold for treatment is lower (higher). Thus, patients treated in high rate regions should have better health at baseline and smaller health gains after treatment. Such a relation would suggest evidence of *unwarranted variations*. Accordingly, the aim of this study is to explore whether the “flat of the curve” phenomenon is present in lumbar spine surgery, and, if demonstrated, to quantify it.

## Methods

Our analysis was based on three linked data sets, collected between 2010 and 2015: administrative registry data from the Norwegian patient registry (NPR), medical registry data from the Norwegian registry for spine surgery (NORspine), and information about patients’ education level from Statistics Norway (SSB). NPR contains information on all patients who have received government-financed specialized care. By law, the NPR is exempt from requiring informed consent at registration.

### Data collection in NORspine

NORspine is a comprehensive medical registry for quality control and research. It receives funding from the government and has no ties to industry. All patients undegoing surgery for degenerative disorders in the lumbar spine are invited to participate in the registry, and consent forms are obtained from all participants. In 2015, NORspine comprised 38 of 40 (93%) public and private hospitals performing surgery for degenerative disorders in the lumbar spine. The case completeness rate was 63% [17].

Upon admission for surgery, patients completed a baseline questionnaire on demographics, lifestyle, and patient-reported HRQoL. During the hospital stay, the surgeon used a standard registration form to record data on diagnosis, treatment, and comorbidity. At 3 and 12 months post-surgery, patients received questionnaire similar to the one completed at baseline via regular post, completed it at home, and returned it in pre-stamped envelopes to the central registry unit. Nonrespondents received one reminder that included a new copy of the questionnaire.

The NORspine protocol has been approved by the Data Inspectorate of Norway. It handled all registration at follow-up without involvement from the treating institution. All patients were granted treatment before answering the questionnaire, and they had no incentive to over- or under-report their true health condition.

### Patient-reported outcome measure

NORspine contains two PROM instruments: the generic EuroQol with 5 dimensions (EQ-5D) and the disease-specific Oswestry Disability Index (ODI). The EQ-5D version used in NORspine describes each dimension along one of 3 levels, yielding 243 possible health-state combinations that are assigned health-state values derived from a population sample in the United Kingdom [18].

The ODI (version 2.1a) includes 10 questions about the limitations of daily living activities. Each item is rated from 0 to 5 and then summarized into a total percentage score ranging from 0 (none) to 100 (maximum pain-related disability) [19]. In the absence of PROM at 12 months, we used last observed carried forward (PROM at 3 months).

### Inclusion, exclusion, and merging

Defined by a selection algorithm developed by NORspine, the sample obtained from NPR was based on diagnosis codes (ICD-10) in combination with procedure codes (NCSP). It included all patients who received publicly-funded surgery for LDH or LSS within our time frame (36,378 observations).

NORspine excludes patients who are: unable or unwilling to submit information; under 16 years of age; have documented drug abuse, severe psychiatric disorders, traumatic or infectious conditions, or; tumors involving the spine. We used NORspine criteria to exclude 860 patients from the NPR sample. Hence, we calculated treatment rates based on 35,518 treatments.

**Fig. 1:**
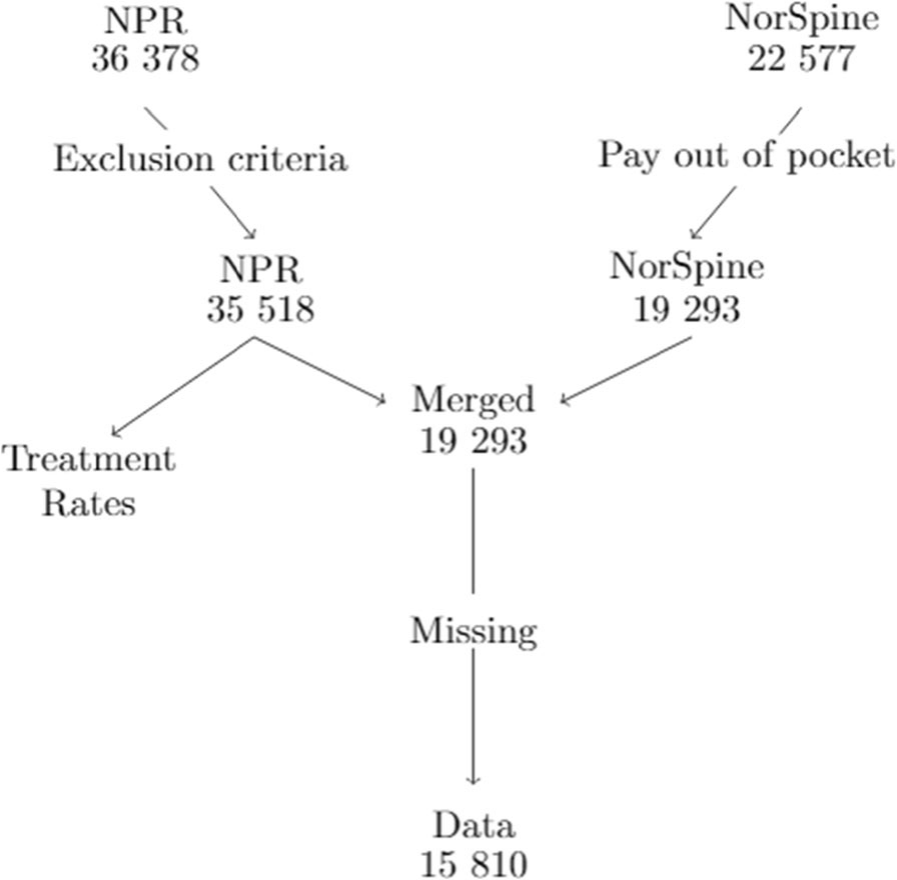
Flow chart of data merging and excluding

Registries were merged based on hospital admission date and an encrypted version of an 11-digit personal identification number. Among 22,577 observations from NORspine, we were unable to match 3284 observations with NPR, largely because NORspine also contains observations on treatments financed out of pocket or by private insurance, which are not part of NPR. We were able to match 19,293 of the observations from NORspine with NPR. After matching, we omitted all observations with missing values for EQ-5D at baseline (1598), smoker status (169), labor market affiliation (315), BMI (944), previous surgery (268), and duration of symptoms (710). The matching proces is illustrated by Fig. 1. Our analysis was based on 15,810 observations (8120 LDH and 7690 LSS).

### Covariates

For statistical estimation, we selected covariates thought to affect patients HRQoL at baseline and health gain. Sociodemographic variables included age (centered at the mean), sex (ref: women), university degree (yes/no, ref.: no), and labor market affiliation (working vs. all alternatives listed as unemployed/sick leave, labor market participation program; retired, permanent disability, homemaker, ref.: working). Health-related behavior include smoker (ref: no) and body mass index (> 30 [obesity] ref.: < 30). Clinical variables included symptoms for longer than 12 months (e.g., pain radiating to legs) (ref: symptoms for less than 12 months); hospital admission (emergency, elective, ref.: elective); previous surgery (no; ‘yes, same, or different level’, ref.: no); and American Acossiation of Anesthesiologist Classification (> = 3, ref.: <=2). We included the following system variables: treated within own hospital area (own hospital service area; own hospital trust but different area; other hospital trust, ref.; own hospital service area); regional effects (19 regions); and time-trend (1:6).

When estimating health gain, we also included duration of hospital stay (days, count). For simplicity, the results reported here include only the coefficients for treatment rates, with health measured by EQ-5D (see Appendix Table A2 and A3 for all coefficients).

### Analysis

We used direct standardization to calculate population treatment rates per 10,000, using publicly available data from SSB to adjust for gender and age composition in each of the 428 Norwegian municipalities.

We used a general estimating equation (GEE) to estimate the relationship between patients’ health and treatment rates [20]. This allowed us to adjust health for individual patient characteristics, account for clustering within regions, and estimate a global effect. We considered using other random- or fixed-effect models, but concluded that a GEE would yield more robust estimates due to data distribution and an unknown correlation structure. To find the best fit for the model, we tested the standard functional forms (linear, polynomials, exponential, and logarithmic). For treatment rates, we used partial derivatives to estimate the marginal effects.

While there is no standardized way to measure the goodness of fit for a GEE model, we applied the method suggested by Zheng [21] in calculating the ${R}_{marg}^2$. We estimated the model with an independence correlation structure and a Gaussian link function. As part of the sensitivity analysis, we excluded patients who received emergency treatment, using only EQ-5D reported at 3 months, or estimated the model using ODI (see Appendix). We conducted the same analysis using regional effects as a random intercept. The association between health and treatment rates concurred with the GEE model, with comparable effect measures. When including regional dummy-variables in a fixed effects model, the results were similar to those in the GEE. Other sensitivity analysis included only regions with a NORspine response rate higher than 20, 30%, or 40%. All sensitivity test results reported here were consistent. All estimations were conducted using R 3.4.0 software (https://www.r-project.org/).

## Results

### Variation in health and utilization rates

Table 1 presents the regions in ascending order with regard to mean annual treatment rates, followed by the NORspine response rate. Subsequent columns show median EQ-5D values at baseline and health gain. Additional file 1: Table A1 in appendix shows the statistics of covariates.

**Table 1:** Surgery rates, median EQ-5D at baseline and health at follow-up, number of Disc and Stenosis patients treated and observed, and number of Disc patients relative to Stenosis patients, by region

	Rates	Responsrate	EQ-5D Base	EQ-5D Gain
Telemark	7,9	22	0,174	0,140
Nordland	8,8	54	0.159	0,396
Fonna	9,0	52	0,189	0,292
Ostfold	9,3	29	0,159	0,309
Oslo Universitetssykehus	10,0	29	0,364	0,209
Finnmark	10,7	59	0,184	0,380
Sorlandet	11,3	52	0,159	0,343
Møre og Romsdal	11,4	39	0,260	0,280
Universitetssykehuset i Nord Norge	11,8	62	0,159	0,413
Bergen	11,9	53	0,189	0,309
Helgeland	12,0	57	0,159	0,413
Innlandet	12,5	48	0,195	0,272
Vestfold	12,5	12	0,159	0,204
St.Olavs	12,9	45	0,159	0,397
Akershus	13,1	32	0,228	0,254
Forde	13,2	22	0,260	0,273
Vestre Viken	13,9	45	0,364	0,223
Stavanger	14,6	60	0,178	0,273
Nord Trondelag	19,0	51	0,159	0,231
**Total**	**11,9**	**43,3**	**0,203**	**0,289**

From Table 1, we computed a variation coefficient by dividing the sum of the three highest rates by the sum of the three lowest rates. The aggregate variation coefficient was 1.85. Considering each year independently, the coefficient ranged from 2.39 (in 2010) to 1.74 (in 2014). The widest range of treatment rates (20.4 in Nord-Trondelag and 6.3 in Telemark) occured in 2010.

At baseline, median EQ-5D varied from 0.159 to 0.364 (interquartile range = 0.053). When considering EQ-5D health gain, the median scores varied from 0.14 to 0.413 (interquartile range = 0.120). Using ANOVA (F-value) and Kurskal-Wallis test (*χ*^2^ value), we found significant variation in EQ-5D between the groups, both at baseline (F = 7,16, *χ*^2^ = 132,29) and health gain (F = 7,91, *χ*^2^ = 131,08).

**Fig. 2:**
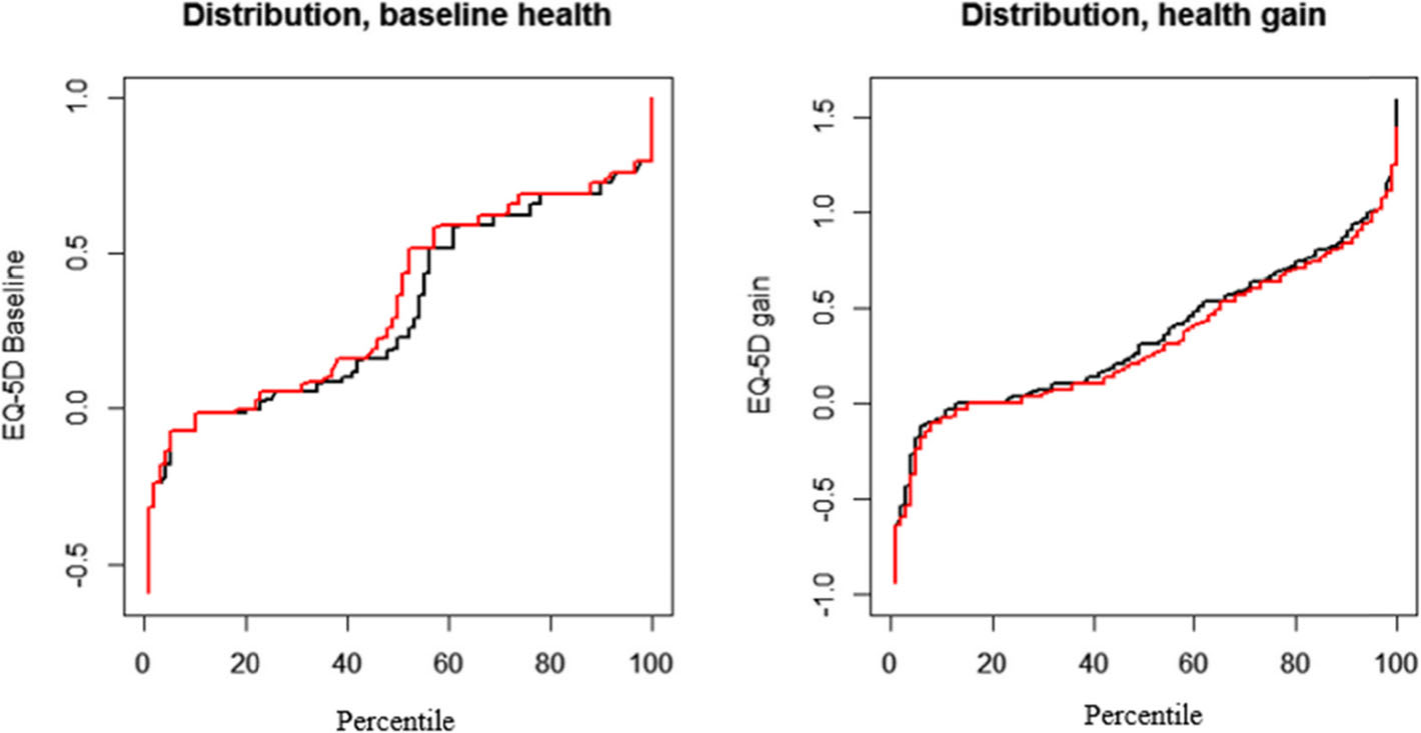
Distribution of health at baseline, and health gain. Black curves represent the three regions with lowest rates, while red curve represent the three regions with highest rates

Figure 2 shows the distribution of unadjusted EQ-5D scores, the distribution for EQ-5D at baseline, and EQ-5D health gain. Even visual inspection of unadjusted EQ-5D scores showed a small but consistent difference in health between the grouped regions. The high-rate regions treated healthier patients and had consistently lower health gains.

### Model output

Table 2 presents the output of the GEE estimation, with significance based on robust standard errors. Linear terms and square roots yielded the best fit of all models. At baseline, we found a positive correlation between EQ-5D and treatment rates, indicating that the average patient was healthier at the time of treatment as treatment rates increased.

**Table 2:** The global effects of treatment rates on baseline health, and health gain measured by EQ-5D

	Baseline health		Health Gain	
	Linear	Best non-linear	Linear	Best non-linear
Intercept	0.353***	0.322***	0.440***	0.495***
Rates	0.002***		−0.004***	
$\sqrt{Rates}$		−0.17***		−0.031***
R^2^_Marg_				
Observations	15,810		12,232	

*p < 0.1;**p < 0.05;***p < 0.01

Adjusted for: treated within or outside own hospital region; age; gender; smoker, BMI; education; labour market participation; previous surgery; emergency care; self-reported measure on duration of symptoms; and time trend. Significance based on robust standard errors

We observed a negative correlation between health gain and treatment rates. Thus, patients’ average health gain decreased as treatment rates increased.

**Fig. 3:**
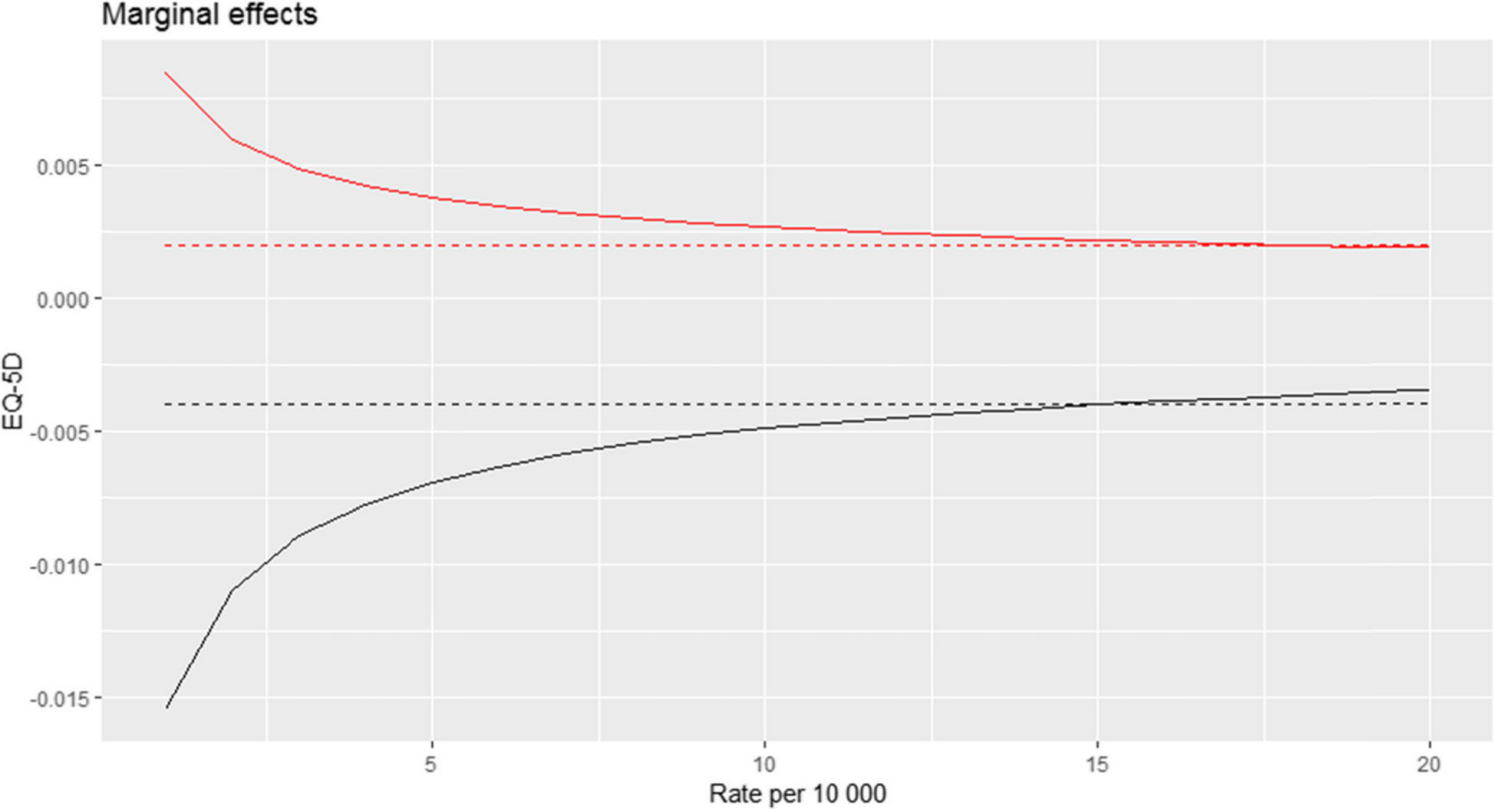
Plotting treatment rates marginal effect on EQ-5D. The two red curves represents EQ-5D at baseline, black curves represents EQ-5D health gain. Stapeled curves are linear models, solid are non-linear models

Figure 3 depicts the marginal effect of treatment rates on EQ-5D. Naturally, the marginal effect from the linear models are constant. For the nonlinear model estimating EQ-5D at baseline, better health was associated with increases in treatment rates, but at a decreasing rate. Similarly, for the marginal effect of treament rates on health gain, increased treatment rates were associated with lower health gain, but at a decreasing rate.

Consequently, given equal patient population characteristics, the EQ-5D baseline score of a patient living in a region with a treatment rate of 8 per 10,000 likely would be 0.024 higher on average, compared to a patient treated in a region with a treatment rate of 18 per 10,000. Given the same two rates, patients in the high-rate region would on average experience 0.044 lower EQ-5D gains than patients in the low-rate region. If we consider the same measures based on ODI, there is no difference at baseline, while the difference in health gain between regions treating 8 or 18 per 10,000 would be 16.31 (See appendix Table A3).

## Discussion

This study shows that, on average, higher treatment rates are associated with better health at baseline and lower health gains. This indicates that unwarranted variations occur in surgical treatment for degenerative lumbar spine disorders, independently of whether we define need as ill health or capacity to benefit. The effect size is moderate, but large enough to display statistically significant contrasts in the mean health of the patients, hence, the marginal effect on a patient level is therefore considerably larger.

The results suggest that patients face different barriers to care, depending on their place of residence. In high rate regions, the average patient’s baseline health is better, and their health gains are lower, confirming the “flat of the curve-phenomenon” The variation is in conflict with a longstanding egalitarian Norwegian health policy, which has ‘equal access for equal need’ as one of it’s specific goals. Place of residence is explicitly stated a factors that should not influence access to health care [21].

Varagunam et al. [2015] considered the relationship between EQ-5D and disease specific PROMS with surgeon volumes for three elective surgeries but found no significant effects [22]. Rachet Jacquet et al. [2019] considered the causal link between hospital volume and patient outcome in hip fractures, and found small but not clinically significant effects [23]. In contrast, the present study considers the population perspective, not the physician perspective. To the best of our knowledge, no previous large-scale studies studies provide the level of detailed HRQoL measures from a population perspective, as we do here. Keller et al. [1999] determined that the concave relationship between treatment rates for LDH affect EQ-5D, both at baseline and health gain [24]. However, that cross-sectional study included only three regions in a US system, with fewer than 500 patients. Our patient-level register data provide a representative sample of the patient population.

Returning to Wennbergs’ three categories of care, when the presence and duration of symptoms are consistent with clinical and imaging findings, there is a high degree of consensus in the medical community about treatment decisions, and patients experience large health gains. Hence, if only such patients were treated, the treatments would likely reflect “effective care”. However, when a patient presents with unspecific symptoms, not obviously consistent with clinical and imaging findings, there might be an ambiguity among specialist about whether or not invasive treatment is beneficial. Table A1 shows large variations in case mix across regions, and Tables A2 and A3 depict how socioeconomic, lifestyle, and clinical factors predict both health at baseline and health gains (Appendix). Education, labor market affiliation, smoking, and body mass index vary markedly in the patient population between regions in our sample. Whether this is an expression of preferences or mirror the general population is unclear. In any case, better knowledge about whether physicians should consider lifestyle factors when considering treatment options, might lead to more similar decision-making processes and reduction of unwarranted variation. Such ambiguity is also present in primary care, and reflected in the rate of patients who are reffered to diagnostic imaging [25].

Due to crowding out effects (a surgeon can only treat one patient at the time), it is impossible to estimate the fraction of variations related to supply effects, without first knowing all activity in a hospital. Even then, it is questionable what yardstick one would use to produce a correct meassure of supply – i.e. surgeons, beds, staff, operation rooms etc. However, it is not unlikely that some of the variation we observe is caused by such supply effects.

Our data do not allow analysis of differences in physicians preferences versus differences in supply as possible causes for the observed regional variation in utilization rates. Variation in preferences are cultural phenomenons, as physicians are quick to adapt their behavior to the enviroment they operate in [26]. Possible approaches to reduce such variation include peer review of practice patterns, such as clinical audits, educational initiatives, development of standardized decision support and leverage of economical incentives, such as the reimbursment per procedure [27]. On the other hand, differences in capacity, such as the number of spine surgeons per population, or surgeons availablity to operating rooms, may cause variation. Possible approaches to reduction of such variation include leadership engagement and action, such as staff recruitment or reduction, and changes in priority between surgical specialties in allocation of operating room capacity. We suggest that comprehensive multi-level analysis of registry data to identify factors associated with variation both on the individual level (patients and surgeons) and group-level, including clustering within units at higher levels (municipalitities, hospitals and health trusts) is necessary to address specific causes for unwarranted variation. Stricter clinical guidelines about indications for surgey and implemeting clinically relevant performance metrics for value-based health care have been suggested to reduce the number of unneccessary and inefficient surgical procedures [28, 29].

### Strengths and weaknesses

The analysis reported here is based on data that is representative for the treated population. Furthermore, our generic and disease-specific HRQoL both yielded similar results.

Range of sensitivity testing did not affect our results. The data do not contain full information on EQ-5D at follow-up. However, a loss to follow-up study found no difference in health between respondents and nonrespondents [30].

Future studies of this subject should include data on the number of patients on waiting lists for treatments, alternatively how long patients waited before receiving care. By inclusion of such data in the analysis, patient specific marginal effects can be estimated. These data were not available for the current study.

## Conclusion

The analysis presented here shows a clear association between increasing treatment rates and better health at baseline, and furthermore, lower health gains, indicating unwarranted wariaions. Our findings confirm the“flat of the curve”-phenomenon on regional basis, indicating conflicts with the Norwegian egalitarian health policy.

## Supplementary information

**Additional file 1.** Table A1

**Additional file 2.** Table A2. Full GEE output. EQ-5D as dependent variable

**Additional file 3.** Table A3. Full GEE output. ODI as dependent variable

## Data Availability

The data that support the findings of this study are available from the NPR and NORspine but restrictions apply to the availability of these data, which were used under license for the current study, and so are not publicly available. Data are however available from the authors upon reasonable request and with permission of The Norwegian Centre for Research Data, The Regional Ethics Committee and NPR.

## Abbreviations

EQ-5D: EuroQol Five-Dimentions
GEE: General estimating equation
HRQoL: Health-related quality of life
LDH: Lumbar disc herniation
LSS: Lumbar spinal stenosis
NORspine: Norwegian Registry for Spine Surgery
NPR: Norwegian Patient Registry
ODI: Oswestry Disability Index
PROM: Patient-reported outcome data
SSB: Statistics Norway

## References

[1] Birkmeyer JD, Reames BN, McCulloch P, Carr AJ, Campbell WB, Wennberg JE (2013). Understanding of regional variation in the use of surgery. Lancet.

[2] Corallo AN, Croxford R, Goodman DC, Bryan EL, Srivastava D, Stukel TA (2014). A systematic review of medical practice variation in OECD countries. Health Policy.

[3] Mercuri M, Birch S, Gafni A (2013). Using small-area variations to inform health care service planning: what do we ‘need’to know?. J Eval Clin Pract.

[4] Our World in Data, Our World in Data. Available: https://ourworldindata.org/grapher/child-mortality-vs-health-expenditure-over-time. [Funnet 5 Febuary 2019].

[5] Our World in Data, Our World in Data. Available: https://ourworldindata.org/grapher/life-expectancy-vs-health-expenditure. [Funnet 5 Febuary 2019].

[6] Fisher ES, Wennberg JE, Stukel TA, Skinner JS, Sharp SM, Freeman JL, Gittelsohn AM (2000). Associations among hospital capacity, utilization, and mortality of US Medicare beneficiaries, controlling for sociodemographic factors. Health Serv Res.

[7] Enthoven AC (1978). Cutting cost without cutting the quality of care. N Engl J Med.

[8] Wennberg JE (2002). Unwarranted variations in healthcare delivery: implications for academic medical centres. BMJ.

[9] Knudsen AK, Kinge JM, Skirbekk V, Vollset SE (2016). Sykdomsbyrde i Norge 1990–2013. Resultater fra Global burden of diseases, injuries, and risk factors study 2013 (GBD 2013).

[10] Brage S, Ihlebaek C, Natvig B, Bruusgaard D (2010). Musculoskeletal disorders as causes of sick leave and disability benefits. Tidsskr Nor Laegeforen.

[11] Tosteson ANA, Tosteson TD, Lurie JD, Abdu W, Herkowitz H, Andersson G, Albert T, Bridwell K, Zhao W, Grove MR, others (2011). Comparative effectiveness evidence from the spine patient outcomes research trial: Surgical vs. non-operative care for spinal stenosis, degenerative spondylolisthesis and intervertebral disc herniation. Spine.

[12] Fisher ES, Wennberg DE, Stukel TA, Gottlieb DJ, Luca FL, Pinder EL (2003). The implications of regional variations in Medicare spending. Part 2: health outcomes and satisfaction with care. Ann Intern Med.

[13] Doyle JJ (2011). Returns to local-area health care spending: evidence from health shocks to patients far from home. Am Econ J Appl Econ.

[14] Johansson N, Jakobsson N, Svensson M (2018). Regional variation in health care utilization in Sweden--the importance of demand-side factors. BMC Health Serv Res.

[15] Birkmeyer JD, Sharp SM, Finlayson SRG, Fishe ES, Wennberg JE (1998). Variation profiles of common surgical procedures. Surgery.

[16] Solberg T, Olsen LR (2016). NORspine Annual Report 2015 [Nasjonalt kvalitetsregister for ryggkirurgi (NKR)]. Årsrapport for 2015 med plan for forbedringstiltak 2016.

[17] Dolan P (1997). Modeling valuations for EuroQol health states. Med Care.

[18] Fairbank JCT (2014). Why are there different versions of the Oswestry disability index?: a review. J Neurosurg Spine.

[19] Zeger SL, Liang K-Y (1986). Longitudinal data analysis for discrete and continuous outcomes. Biometrics.

[20] Zheng B (2000). Summarizing the goodness of fit of generalized linear models for longitudinal data. Stat Med.

[21] Norheim O, Allgott B, Aschim B, Førde R, Gjul G, Gundersen T, Kakad MKA, Kvinnsland S, Melberg H, others (2014). Åpen og rettferdig- prioritering i helsetjenesten [Open and fair - priority serring in the health service], Official Norwegian Reports 2014:12.

[22] Varagunam M, Hutchings A, Black N (2015). Relationship between patient-reported outcomes of elective surgery and hospital and consultant volume. Med Care.

[23] Rachet Jacquet L, Gutacker N, Siciliani L (2019). The causal effect of hospital volume on health gains from hip replacement surgery.

[24] Keller RB, Atlas SJ, Soule DN, Singer DE, Deyo RA (1999). Relationship between rates and outcomes of operative treatment for lumbar disc herniation and spinal stenosis. JBJS.

[25] Gransjøen AM, Lysdahl KB, Hofmann BM (2019). Geographical variations in the use of diagnostic imaging of musculoskeletal diseases in Norway. Acta Radiologica.

[26] Molitor D (2018). The evolution of physician practice styles: evidence from cardiologist migration. Am Econ J Econ Pol.

[27] Holtedahl R, Brox JI, Aune AK, Nguyen D, Risberg MA, Tjomsland O (2018). Changes in the rate of publicly financed knee arthroscopies: an analysis of data from the Norwegian patient registry from 2012 to 2016. BMJ Open.

[28] Brownlee S, Chalkidou K, Doust J, Elshaug AG, Glasziou P, Heath I, Nagpal S, Saini V, Srivastava D, Chalmers K, others (2017). Evidence for overuse of medical services around the world. Lancet.

[29] Miller G, Rhyan C, Beaudin-Seiler B, Hughes-Cromwick P (2018). A framework for measuring low-value care. Value Health.

[30] Solberg TK, Sorlie A, Sjaavik K, Nygaard OP, Ingebrigtsen T (2011). Would loss to follow-up bias the outcome evaluation of patients operated for degenerative disorders of the lumbar spine? A study of responding and non-responding cohort participants from a clinical spine surgery registry. Acta Orthopaedica.

