# Supplementary material for: Associations between utilization rates and patients’ health: a study of spine surgery and patient-reported outcomes (EQ-5D and ODI)
Source: BMC Health Serv Res. 2020 Feb 22;20:135. doi: 10.1186/s12913-020-4968-2 (PMC7036171; doi:10.1186/s12913-020-4968-2)
Supplement: Supplementary file 1 — Additional file 1. Table A1 [file 12913_2020_4968_MOESM1_ESM.docx]

|  | Helgeland | Telemark | Nordland | Fonna | Ostfold | OUS | Finnmark | Sorlandet | MogRomsdal | UNN | Bergen | Innlandet | Vestfold | St.Olavs | Akershus | Forde | VViken | Stavanger | NTrondelag | Mean | Min | Max |
| --- | --- | --- | --- | --- | --- | --- | --- | --- | --- | --- | --- | --- | --- | --- | --- | --- | --- | --- | --- | --- | --- | --- |
| Age | 57 | 57 | 54 | 54 | 53 | 57 | 51 | 55 | 54 | 51 | 54 | 55 | 54 | 54 | 56 | 55 | 57 | 54 | 54 | 54,52632 | 51 | 57 |
| Women | 45,24 | 42,19 | 44,05 | 48,61 | 51,13 | 49,04 | 40,85 | 46,2 | 49,37 | 39,74 | 46,82 | 48,26 | 48,34 | 44,64 | 52,67 | 45,54 | 50,91 | 45,58 | 48,23 | 46,70579 | 39,74 | 52,67 |
| ASA <= 2 | 85,98 | 76,6 | 87,37 | 91,23 | 88,73 | 88,69 | 90,71 | 82,45 | 85,47 | 92,82 | 92,61 | 87,43 | 87,98 | 85,2 | 89,43 | 93 | 88,83 | 91,85 | 90,33 | 88,24789 | 76,6 | 93 |
| Smokers | 24,4 | 32,81 | 24,56 | 31,73 | 29,16 | 22,3 | 34,86 | 27,68 | 24,68 | 24,87 | 26,14 | 29,28 | 27,01 | 24,18 | 24,4 | 21,78 | 23,38 | 24 | 26,89 | 26,53211 | 21,78 | 34,86 |
| Emergency | 19,94 | 3,65 | 24,3 | 11,5 | 4,31 | 4,24 | 24,3 | 9,94 | 8,74 | 29,87 | 9,51 | 4,92 | 5,69 | 19,87 | 4,61 | 9,41 | 5,96 | 19,58 | 16,16 | 12,44737 | 3,65 | 29,87 |
| High educ | 18,45 | 23,44 | 25,06 | 20,78 | 24,02 | 42,89 | 28,52 | 24,76 | 22,71 | 29,36 | 25,37 | 18,65 | 25,12 | 29,46 | 23,87 | 20,79 | 28,81 | 22,3 | 20,08 | 24,97053 | 18,45 | 42,89 |
| Obese | 25,89 | 29,69 | 25,82 | 25,79 | 25,05 | 18,57 | 22,18 | 23,1 | 23,55 | 21,92 | 22,88 | 24,89 | 25,59 | 23,81 | 26,91 | 17,33 | 23,5 | 22,91 | 30,43 | 24,20053 | 17,33 | 30,43 |
| Prev Surg | 28,27 | 19,79 | 22,03 | 27,83 | 31,83 | 25,43 | 22,89 | 23,2 | 24,4 | 21,79 | 27,15 | 25,94 | 26,07 | 29,09 | 27,02 | 32,18 | 23,67 | 31,58 | 35,61 | 26,61947 | 19,79 | 35,61 |
| Own Region | 27,38 | 40,62 | 12,41 | 22,63 | 19,1 | 74,67 | 0 | 87,43 | 80,11 | 86,28 | 98,69 | 83,91 | 28,44 | 98,66 | 29,63 | 10,4 | 81,94 | 95,39 | 81,57 | 55,75053 | 0 | 98,69 |
| Own Trust | 33,63 | 54,17 | 60 | 75,32 | 78,03 | 23,21 | 82,75 | 7,02 | 16,93 | 0,13 | 0,24 | 12,87 | 66,35 | 0,3 | 67,96 | 79,21 | 14,55 | 3,64 | 15,28 | 36,39947 | 0,13 | 82,75 |
| Other Trust | 38,99 | 5,21 | 27,59 | 2,04 | 2,87 | 2,12 | 17,25 | 5,56 | 2,96 | 13,59 | 1,07 | 3,22 | 5,21 | 1,04 | 2,41 | 10,4 | 3,51 | 0,97 | 3,16 | 7,851053 | 0,97 | 38,99 |
| Working | 11,61 | 10,94 | 15,95 | 16,51 | 13,35 | 20,69 | 18,31 | 15,3 | 16,64 | 17,69 | 16,16 | 14,38 | 15,17 | 18,08 | 17,8 | 18,32 | 19,17 | 22,24 | 15,28 | 16,50474 | 10,94 | 22,24 |
| Sick leave | 43,75 | 41,67 | 50,13 | 42,12 | 49,69 | 37,54 | 52,46 | 41,52 | 48,24 | 54,74 | 46,41 | 43,53 | 40,76 | 45,83 | 42,2 | 49,5 | 40,27 | 40,36 | 45,2 | 45,04842 | 37,54 | 54,74 |
| Outside Labor force | 44,64 | 47,4 | 33,92 | 41,37 | 36,96 | 41,78 | 29,23 | 43,18 | 35,12 | 27,56 | 37,43 | 42,09 | 44,08 | 36,09 | 40 | 32,18 | 40,56 | 37,39 | 39,52 | 38,44737 | 27,56 | 47,4 |
| sym < 12 months | 49,11 | 35,42 | 54,94 | 51,58 | 44,35 | 44,6 | 59,15 | 51,66 | 52,19 | 63,33 | 59,18 | 50,23 | 44,55 | 61,24 | 47,75 | 50,5 | 49,97 | 56,91 | 55,56 | 51,69579 | 35,42 | 63,33 |
