# Supplementary material for: Associations between utilization rates and patients’ health: a study of spine surgery and patient-reported outcomes (EQ-5D and ODI)
Source: BMC Health Serv Res. 2020 Feb 22;20:135. doi: 10.1186/s12913-020-4968-2 (PMC7036171; doi:10.1186/s12913-020-4968-2)
Supplement: Supplementary file 2 — Additional file 2. Table A2. Full GEE output. EQ-5D as dependent variable [file 12913_2020_4968_MOESM2_ESM.docx]

*Table A2: Full GEE output. EQ-5D as dependent variable*

|  | EQ-5D Base | | | EQ-5D Gain | | |
| --- | --- | --- | --- | --- | --- | --- |
|  | Linear | Non-linear | | Linear | Non-Linear | |
| Rates | 0.002^∗∗^ |  | | −0.004^∗∗^ |  |  |
| *Rates* |  | 0.017^∗∗^ | |  | −0.031^∗∗^ | |
| LSS | 0.071^∗∗∗^ | 0.071^∗∗∗^ | | −0.101^∗∗∗^ | −0.101^∗∗∗^ | |
| Age | 0.0004 | 0.0004 | | 0.0002 | 0.0002 | |
| Male | 0.063^∗∗∗^ | 0.063^∗∗∗^ | | −0.030^∗∗∗^ | −0.030^∗∗∗^ | |
| Emergency | −0.236^∗∗∗^ | −0.236^∗∗∗^ | | 0.222^∗∗∗^ | 0.222^∗∗∗^ | |
| Own Trust | −0.013 | −0.013 | | 0.002 | 0.002 | |
| Other Trust | 0.002 | 0.003 | | −0.00001 | −0.0002 | |
| ASA < 3 | 0.049^∗∗∗^ | 0.049^∗∗∗^ | | 0.009 | 0.009 | |
| Smoke | −0.031^∗∗∗^ | −0.031^∗∗∗^ | | −0.044^∗∗∗^ | −0.044^∗∗∗^ | |
| Previous surgery | −0.066^∗∗∗^ | −0.066^∗∗∗^ | | −0.048^∗∗∗^ | −0.048^∗∗∗^ | |
| BMI>30 | −0.021^∗∗∗^ | −0.021^∗∗∗^ | | −0.015^∗^ | −0.015^∗^ | |
| Sick leave | −0.161^∗∗∗^ | −0.161^∗∗∗^ | | 0.082^∗∗∗^ | 0.082^∗∗∗^ | |
| Outside laborforce | −0.157^∗∗∗^ | −0.157^∗∗∗^ | | 0.047^∗∗∗^ | 0.047^∗∗∗^ | |
| Higher educ | 0.031^∗∗∗^ | 0.031^∗∗∗^ | | 0.029^∗∗∗^ | 0.029^∗∗∗^ | |
| T-trend | −0.002 | −0.002 | | −0.001 | −0.001 | |
| Sympt > 12 months | 0.004 | 0.004 | | −0.092^∗∗∗^ | −0.092^∗∗∗^ | |
| Constant | 0.353^∗∗∗^ | 0.322^∗∗∗^ | | 0.440^∗∗∗^ | 0.495^∗∗∗^ | |
| Observations | 15,810 | | 15,810 | 12,232 | | 12,232 |

*Note:* ^∗^p*<*0.1; ^∗∗^p*<*0.05; ^∗∗∗^p*<0.01*
