# Supplementary material for: Associations between utilization rates and patients’ health: a study of spine surgery and patient-reported outcomes (EQ-5D and ODI)
Source: BMC Health Serv Res. 2020 Feb 22;20:135. doi: 10.1186/s12913-020-4968-2 (PMC7036171; doi:10.1186/s12913-020-4968-2)
Supplement: Supplementary file 3 — Additional file 3. Table A3. Full GEE output. ODI as dependent variable [file 12913_2020_4968_MOESM3_ESM.docx]

*Tab A3: Full GEE output. ODI as dependent variable*

ODI base ODI gain

Linear Non-Linear Linear Non-Linear

| Rates | −0.085 |  | −0.222^∗∗^ |  |
| --- | --- | --- | --- | --- |
| $\sqrt{Rates}$ |  | −0.687 |  | −1.631^∗∗^ |
| LSS | −5.198^∗∗∗^ | −5.198^∗∗∗^ | −6.926^∗∗∗^ | −6.923^∗∗∗^ |
| Age | 0.053^∗∗∗^ | 0.053^∗∗∗^ | 0.036^∗∗^ | 0.036^∗∗^ |
| Male | −4.852^∗∗∗^ | −4.852^∗∗∗^ | −1.816^∗∗∗^ | −1.816^∗∗∗^ |
| Emergency | 15.892^∗∗∗^ | 15.891^∗∗∗^ | 16.269^∗∗∗^ | 16.266^∗∗∗^ |
| Own trust | 0.387 | 0.365 | −0.522 | −0.547 |
| Other Trust | −1.089^∗∗^ | −1.104^∗∗^ | 0.024 | 0.010 |
| ASA < 3 | −3.622^∗∗∗^ | −3.619^∗∗∗^ | −0.518 | −0.517 |
| Duration of stay |  |  | −0.258^∗∗∗^ | −0.258^∗∗∗^ |
| Smoke | 1.727^∗∗∗^ | 1.726^∗∗∗^ | −1.564^∗∗∗^ | −1.566^∗∗∗^ |
| Prev. surg | 3.698^∗∗∗^ | 3.700^∗∗∗^ | −3.336^∗∗∗^ | −3.337^∗∗∗^ |
| BMI > 30 | 1.757^∗∗∗^ | 1.758^∗∗∗^ | −0.244 | −0.245 |
| Sick leave | 9.008^∗∗∗^ | 9.006^∗∗∗^ | 3.449^∗∗∗^ | 3.447^∗∗∗^ |
| Outside labor force | 9.333^∗∗∗^ | 9.330^∗∗∗^ | 2.840^∗∗∗^ | 2.838^∗∗∗^ |
| Higher educ | −1.682^∗∗∗^ | −1.684^∗∗∗^ | 1.366^∗∗∗^ | 1.366^∗∗∗^ |
| T-trend | 0.010 | 0.013 | −0.029 | −0.025 |
| Sym > 12 months | −1.967^∗∗∗^ | −1.969^∗∗∗^ | −6.844^∗∗∗^ | −6.846^∗∗∗^ |
| Constant | 43.218^∗∗∗^ | 44.566^∗∗∗^ | 30.132^∗∗∗^ | 33.088^∗∗∗^ |
| Observations | 15,609 | 15,609 | 12,719 | 12,719 |

*Note:* ^∗^p*<*0.1; ^∗∗^p*<*0.05; ^∗∗∗^p*<*0.01
